# Eye-tracking during free visual exploration of familiar dramatic character faces facilitates rapid and accurate stroke recognition

**DOI:** 10.3389/fnins.2025.1692719

**Published:** 2026-01-06

**Authors:** Qingya Lu, Yimeng Zeng, Xu Wang, Yiwen Chen, Jingyuan Deng, Cong Yan

**Affiliations:** 1 School of Life Sciences, Beijing University of Chinese Medicine, Beijing, China; 2 School of Management, Beijing University of Chinese Medicine, Beijing, China; 3 School of Chinese Materia Medica, Beijing University of Chinese Medicine, Beijing, China; 4 The First Affiliated Hospital of Xi’an Jiaotong University, Xi’an, Shaanxi, China

**Keywords:** eye movement, fixation, saccade, scanpath, stroke, stroke recognition

## Abstract

**Background:**

Stroke patients often experience significant impairments, making rapid and accurate detection crucial for timely intervention and early warning. However, existing diagnostic methods such as advanced neuroimaging are often time-consuming, highly dependent on operator expertise, or costly and complex to deploy, limiting their scalability in resource-restricted settings. Eye movement patterns in stroke patients present a promising opportunity for efficient detection, given their close ties to underlying neurocognitive mechanisms and potential diagnostic sensitivity. Nevertheless, the lack of a feasible task paradigm and robust detection strategy has hindered the practical application of eye movement-based stroke identification. This study aimed to capture eye movement dysfunction associated with stroke through an ecological paradigm and develop a machine learning model with improved diagnostic accuracy.

**Methods:**

We recorded eye movement signals in stroke patients (*N* = 16) and healthy controls (*N* = 23) during free visual exploration of familiar dramatic character faces. A diverse set of eye movement features, encompassing saccadic, fixation, and scanpath features, was extracted and analyzed. These features were subsequently employed to construct machine learning models for the recognition of stroke patients.

**Results:**

We identified distinctive eye movement patterns in the stroke group, including prolonged fixation duration, restricted saccadic movements, and reduced scanpath length, which reflect underlying visual processing impairments. Furthermore, by integrating these multi-dimensional eye movement features, our machine learning model achieved a high accuracy of 87.18% and an excellent area under the receiver operating characteristic curve (AUROC) of 0.92 in distinguishing stroke patients.

**Discussion:**

This study demonstrates that ecologically valid eye-tracking, combined with multi-type feature analytics, serves as a practical screening tool with the potential to significantly improve identification accuracy and alleviate logistical burdens in community and primary care settings.

## Graphical abstract

Eye movement patterns offer a promising opportunity for efficient stroke detection due to their link to neurocognitive mechanisms. Based on this correlation, we develop an ecological paradigm, free visual exploration of familiar dramatic character faces, to capture eye movement dysfunctions in stroke patients. By leveraging multi type eye movement features, we can facilitate rapid and accurate stroke recognition. 
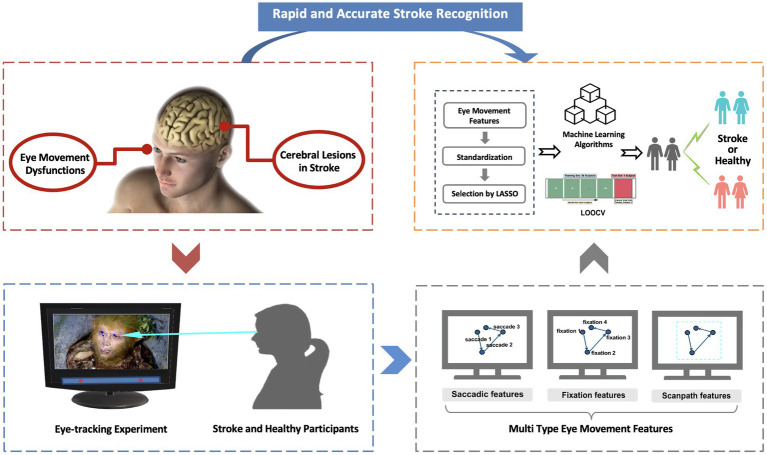

## Introduction

Stroke is a leading global health burden, ranking as the second leading cause of death and the third leading cause of disability-adjusted life-years worldwide, with approximately 12.2 million new cases reported annually (Saver and Levine, 2010). The rapid and early assessment of stroke is paramount, as substantial evidence suggests that therapeutic interventions are most effective when implemented within a critical window shortly after stroke onset (Guo and Saver, 2025; Saenger and Christenson, 2010). Delays in diagnosis can lead to irreversible neurological damage, increased disability burden, and reduced efficacy of rehabilitative therapies (Biernaskie et al., 2004; Salter et al., 2006). Current diagnostic approaches, including neuroimaging techniques such as Computed Tomography (CT) and Magnetic Resonance Imaging (MRI) exhibit high sensitivity in identifying subtle neurological deficits (Powers et al., 2019). However, these methods rely on specialized equipment, involving time-consuming procedures and incurring considerable costs, which restrict their accessibility in community and primary care settings (Chalela et al., 2007; Albers et al., 2018). Although neurocognitive assessments, such as the NIH Stroke Scale (NIHSS), are widely used to evaluate stroke severity and functional outcomes, requiring trained personnel and standardized protocols (Hills et al., 2009). The limited availability of these tools underscores the urgent need for cost-effective and scalable screening methods that facilitate stroke identification, thereby addressing critical logistical challenges.

Oculomotor dysfunction serves as a promising biomarker for stroke, reflecting disruptions in distributed neural networks that are vulnerable to stroke lesions (Kelts, 2010). These networks encompass cortical areas such as the frontal eye fields (FEF) and posterior parietal cortex, along with subcortical regions (van Wyk et al., 2016; Schiller et al., 1980). Stroke affecting these circuits can lead to direct oculomotor deficits, including gaze palsy, nystagmus, and saccadic abnormalities, or can indirectly influence eye movements by impairing attention, spatial cognition, and executive control (Hepworth et al., 2016). For instance, unilateral spatial neglect, frequently observed following a right hemisphere stroke, typically involves a rightward fixation bias and altered saccadic patterns, such as reduced saccade amplitudes during visual exploration, even in patients without overt deficits on standard tests (Delazer et al., 2018). Disruptions in the FEF circuit due to stroke can impair the control of voluntary saccades, which is manifested by significantly increased saccade latency and reduced amplitude (Tehovnik et al., 2000; Dash et al., 2018). Meanwhile, cerebellar stroke commonly disrupts smooth pursuit function, resulting in decreased tracking gain and an increased frequency of catch-up saccades (Bodranghien et al., 2016). Additionally, increased latency in vertical saccadic eye movements and longer fixation durations have been correlated with cognitive impairment post-stroke (Walle et al., 2019; Ionescu et al., 2023). Given the established link between oculomotor dysfunction and stroke, eye tracking technology emerges as a powerful tool for the quantitative assessment of these subtle abnormalities, serving as a valuable complement to traditional neurocognitive assessments (Tao et al., 2020; Kumar et al., 2016; Wolf et al., 2023; Wolf and Ueda, 2021; Zhang et al., 2025).

However, the assessment of oculomotor dysfunction in neurological disorders presents a practical challenge, as patients with cognitive impairments may struggle to follow the instructions of certain eye-tracking paradigms, which undermines measurement validity (de Souza et al., 2023). To mitigate this issue, the free visual exploration (FVE) paradigm employs tasks with minimal instructions to capture natural viewing behavior, thereby reducing cognitive load and minimizing instructional bias (Huang et al., 2024). In particular, facial stimuli have been extensively utilized in FVE and related paradigms due to their significant biological and social relevance (Galambos et al., 2018). The processing of facial features, including aspects of identity and expression, elicits eye movement patterns that provide valuable insights into cognitive function (Henderson et al., 2005). Moreover, to enhance ecological validity, recent studies have utilized static frames from films or television series as experimental stimuli, capitalizing on their retained social and narrative context as well as participants’ pre-existing familiarity with the content to foster automatic processing and promote sustained attentional engagement (Risko et al., 2012; Sonkusare et al., 2019). Nevertheless, the translational potential of using FVE with such dramatic facial stimuli for sensitive functional assessment in stroke populations remains to be established.

**Figure 1:**
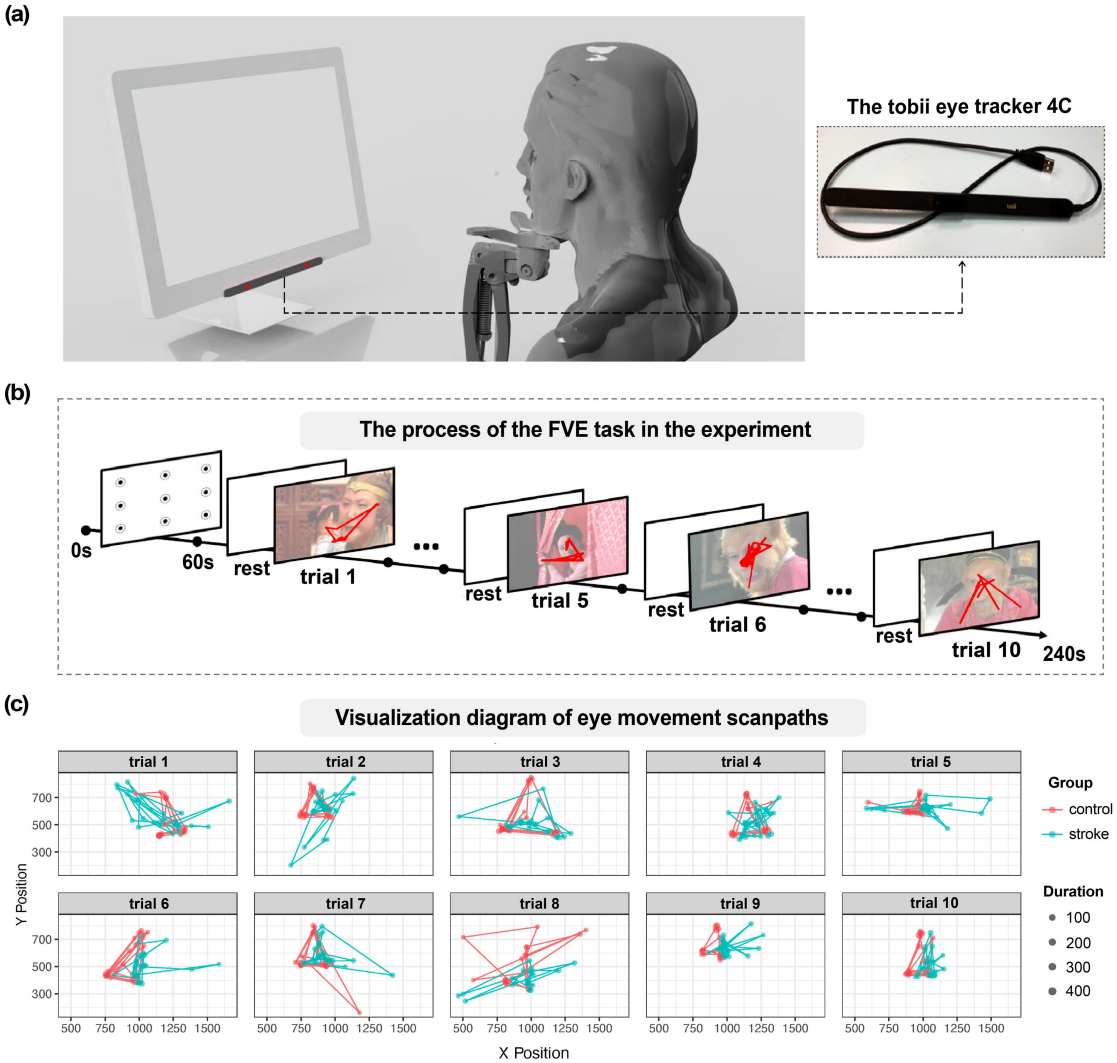
**(a)** Schematic diagram of the eye movement experiment. **(b)** The process of the FVE task of familiar dramatic character faces. **(c)** Visualization of eye movement scanpaths for two subjects, one from the healthy group and one from the disease group, across all face images.

In this study, we propose a lightweight and ecologically valid approach that incorporates facial images of familiar dramatic characters within a FVE paradigm. Recognizing the well-established association between oculomotor dysfunction and stroke pathology, we extracted and analyzed a diverse range of eye movement features, including saccadic, fixation, and scanpath features, to capture the multifaceted nature of neural and cognitive impairments in stroke patients (Figure 1). We hypothesized that the integration of these diverse eye movement features would facilitate the development of a cost-effective and auxiliary screening tool capable of identifying eye movement abnormalities, thereby enhancing accessibility to accurate stroke recognition. Ultimately, our modeling framework aims to establish a novel and complementary pathway for the detection of stroke-related neurological dysfunction through the utilization of interpretable eye movement features.

## Methods

### Participants

This cross-sectional prospective study recruited stroke participants with diverse lesion types and areas from the First Affiliated Hospital of Xi’an Jiaotong University, as well as healthy controls via social media. Stroke participants underwent standard physical, neurological, and imaging exams (CT and MRI) and met Chinese guidelines for acute ischemic stroke (2018) (Chinese Society of Neurology, C.S.S, 2018) and intracerebral hemorrhage (2019) (Society, C.S.o.N.C.S, 2019). Exclusion criteria included neglect, severe visual impairments (e.g., color blindness), fever, brain trauma, cancer, anemia, and neuropsychiatric disorders. Healthy controls had no history of stroke, nervous system diseases, chronic illnesses, major medical conditions, neuro-psychiatric disorders, or vision/hearing abnormalities. All participants provided informed consent, and the study was approved by the Ethics Committee of the Medical College of Xi’an Jiaotong University (No. 2021-687). This study was conducted in strict compliance with the Declaration of Helsinki.

### Procedure

All experiments were conducted on a 23.8-inch display with a resolution of 1920 × 1,080 pixels. Each participant was seated in front of the monitor, with a chin-rest used to stabilize their head and maintain 70 cm between the participants’ eyes and the monitor. A Tobii Eye Tracker 4C (90 Hz sampling rate, ~0.5–1° spatial accuracy) was positioned at the bottom of the screen to record all eye movement data. The experiment took place in a sound-insulated room within the hospital.

The visual stimuli designed to evoke eye movement signals consisted of face images of characters from “*A Dream of Red Mansions*” and “*Journey to the West*.” Participants were instructed to engage in free viewing of the images and to concurrently focus their attention on them, which helped reduce noise from variable engagement levels. After a 9-point calibration of the eye movement signal, each face image from the television series was then presented sequentially for 10 s, preceded by a 5-s grey image. The 10-s presentation duration was chosen based on three critical reasons. First, previous studies indicate that viewing durations range from 2 to 10 s depending on the eye-tracking task complexity (Sonkusare et al., 2019; Frank et al., 2023). Subsequently, this parameter was refined based on feedback obtained from preliminary tests conducted with healthy older adults, as durations below 5 s were found to be inadequate for complete visual exploration, whereas 8–10 s were sufficient. In addition, for stroke patients, longer durations are generally needed to ensure adequate data quality, but excessively long presentations risk distraction with diminishing returns in information gain (Josephson and Holmes, 2002). Consequently, a 10-s duration was selected for the experiment.

To ensure ethical compliance and data quality, participants were informed that they could close their eyes and rest at any time if they felt uncomfortable, which would immediately pause the experiment. The session would be halted until the participant felt comfortable to continue, at which point the experiment would resume from the point of interruption rather than restarting from the beginning, avoiding any re-exposure to prior stimuli. Before resumption, a full 9-point recalibration of the eye-tracking system would be conducted to correct for potential gaze drift and maintain data accuracy. Throughout the session, an experimenter monitored participants for behaviors such as frequent eye closures or signs of fatigue to enable timely intervention, and excluded these participants from the final dataset. However, no participants required a pause or exhibited significant discomfort during the study.

### Data preprocessing

Eye movement data were exported from eye-tracking software. The missing gaze data with gaps shorter than 75 milliseconds(ms) were filled using linear interpolation (Wang et al., 2020). The missing gaze data caused by blink artifacts were removed. And we also removed the data of a trial if its missing gaze data exceeded 20%, because we considered that the participant’s inattention or external interference affected the quality of the eye movement data in that trial.

### Eye movement features extraction

In daily visual behavior, oculomotor nerve activities primarily encompass saccadic and fixation movements (Manor and Gordon, 2003). Saccadic movements facilitate visual system in surveying the environment and processing information (Graupner et al., 2011). These movements direct relevant information glimpsed by the eyes into the fovea, where visual acuity is highest, while fixation behavior allows for a more detailed processing of this information (Huber-Huber et al., 2021). The scanpaths were obtained by time-ordered saccades and fixations, reflecting the relevant characteristics of the overall eye movement intuitively (Schwetlick et al., 2023). Based on this study (Holland and Komogortsev, 2011), we calculated fixation features, saccadic features, and the overall scanpath features:

1. The saccadic features were characterized by *mean vectorial saccade velocity* (degrees per second, deg/s; the average speed of all saccades), *mean vectorial saccade amplitude* (deg; the average distance by each saccade), *inflection count* (the number of directional changes, including vertical and horizontal sequences), *mean vectorial saccade peak velocity* (deg/s; the average of the highest saccadic velocity during each saccade), and *velocity waveform indicator* (the regression slope between peak saccadic velocity and mean saccadic velocity).
2. The fixation features included *mean fixation duration* (ms; the average fixation duration), *fixation count* (the total number of fixations), and *regions of interest* (the number of distinct spatial regions receiving concentrated attention).
3. With regard to scanpath features, we calculated *Shannon entropy* to assess between-group differences in gaze dispersion across various task conditions; higher entropy values indicate greater irregularity in an individual’s scanpath pattern (Vasilyev and Hansard, 2019). Additionally, we computed *scanpath length* (deg) and *scanpath area* (deg^2^), which, respectively, represent the length and coverage area of the scanpath.

### Eye movement intra-individual stability and inter-individual consistency

To assess individual stability (intra-individual) and group-level pattern consistency (inter-individual), we quantified scanpath similarity as a measure of voluntary visual exploration behavior. We examined both within-subject variations when individuals viewed different images and between-group differences when stroke patients and controls were presented with identical stimuli. The scasim function from the R package ‘scanpath’ (version 1.06) (von der Malsburg and Vasishth, 2011) was employed to calculate scanpath similarity by comparing fixation sequences based on their spatial distribution and temporal characteristics, with higher similarity scores approaching 1 indicating more consistent eye movement patterns.

### Data reliability assessment

The free-viewing paradigm, while providing naturalistic observation data, may limit the interpretability of gaze patterns because the absence of a specific task makes it difficult to determine whether observed fixations reflect meaningful cognitive or attentional states or merely idiosyncratic viewing behavior (Velichkovsky et al., 2019). Furthermore, a relatively short total testing duration (approximately 4 min) could affect the reliability and stability of the recorded oculomotor metrics.

**Figure 2:**
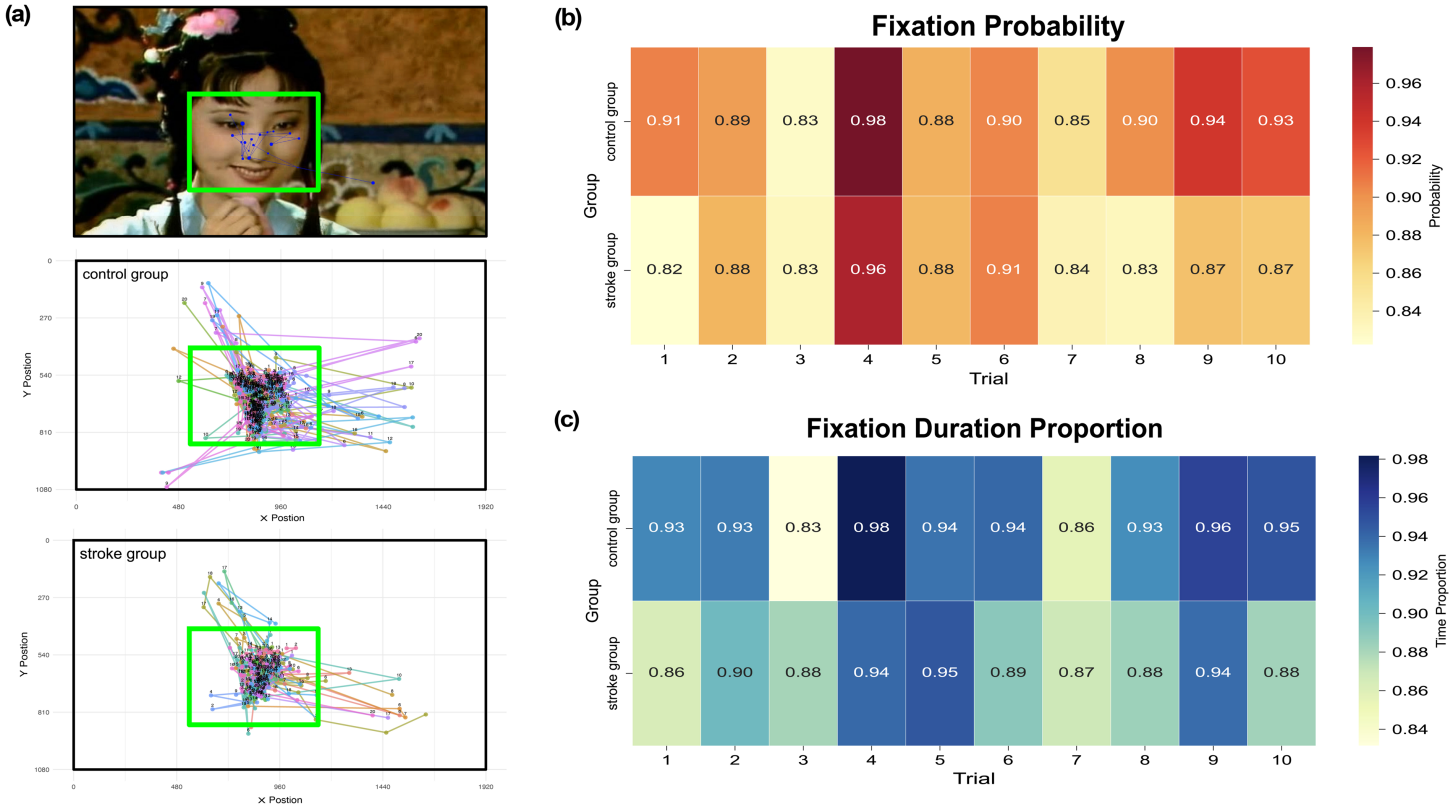
Gaze behavior and AOI analysis. **(a)** Representative stimulus image with a standardized rectangular AOI (solid green line) delineating the core facial features (eyes, nose, mouth). The middle and bottom panels show the aggregate scanpaths of all participants from the healthy control group and stroke patient group, respectively, for the same trial. Each unique color corresponds to an individual participant, with numbered nodes indicating the temporal sequence of fixations. Fixations were predominantly clustered within the defined AOI, with sparse distribution in the background regions. **(b)** Mean proportion of fixations located inside the AOI relative to the total number of fixations within each trial. **(c)** Mean proportion of total fixation duration within the AOI relative to the total fixation duration within each trial.

To address these fundamental concerns about the experimental design and ensure the reliability of the gaze data, we implemented two assessment approaches. Firstly, to examine whether the observed gaze behavior reflects meaningful attention to facial features rather than random or disengaged viewing, we defined a rectangular area of interest (AOI) covering the core facial regions (eyes, nose, and mouth) (Iskra and Gabrijelčič, 2016; Jongerius et al., 2021). The AOI was set to 600 × 450 pixel^2^ to ensure that all key facial areas in every trial were enclosed (Figure 2a). We then calculated the average proportion of fixation points falling inside the AOI and the average proportion of total fixation time inside the AOI.

Secondly, given the limited total testing time, we evaluated the internal consistency of key oculomotor metrics using intraclass correlation coefficients (ICCs) for assessing measurement reliability (Koo and Li, 2016). We computed ICCs across different trial subsets (the first 3, 5, and 7 trials) to estimate the stability of measurements when the number of trials is restricted.

### Statistics analysis

All statistical analyses were conducted using IBM SPSS Statistics software (Version 26.0; IBM Corp.). Initially, the normality of the data distribution for each eye movement feature was assessed using the Shapiro–Wilk test. Based on the results of this normality test, appropriate statistical tests were applied: Independent two-sample t-tests were utilized for normally distributed data to compare eye movement features between the healthy control group and the stroke patient group, while the Mann–Whitney U test was employed for non-normally distributed data. A two-tailed significance threshold of *p* < 0.05 was established to identify statistically significant differences between the groups.

### Machine learning strategy for stroke recognition

To prevent data leakage, all eye movement features from each subject were organized into multiple columns by trial for feature selection and model training. Feature selection methods include filter, wrapper, and embedded approaches (e.g., LASSO) (Huang, 2015). LASSO shrinks irrelevant coefficients to zero, producing sparse and interpretable models. For this study, we implemented LASSO for feature selection using the Scikit-Learn library (Python 3.10), selecting predictive features while discarding redundancies to reduce dimensionality. This dimensionality reduction approach prepares the optimized feature set for subsequent machine learning modeling.

Machine learning employs mathematical models and algorithms to iteratively enhance predictive performance, commencing with a training dataset where the model learns feature-label mappings before being evaluated on unseen test data for accuracy (Andaur Navarro et al., 2022). For our supervised learning framework, we selected 4 complementary algorithms including Support Vector Machine (SVM), Extra Trees, XGBoost, and CatBoost. The model’s performance was evaluated using leave-one-out cross-validation (LOOCV), in which each sample was used once as the test set while the remaining samples served as the training set. Performance assessment was conducted using four key metrics: accuracy, F1-score, area under the receiver operating characteristic curve (AUROC), and area under the precision-recall curve (AUCPR).

## Results

### Group differences of eye movement features

A total of 39 eligible participants were included in the study: 16 stroke patients (10 males, 6 females; mean age ± SD = 61.31 ± 6.59) and 23 healthy controls (13 males, 10 females; mean age ± SD = 62.77 ± 4.65). Five stroke patients were excluded for being unwilling or unable to complete the experiment. The stroke cohort exhibited mild to moderate neurological deficits, as indicated by NIHSS scores ranging from 3 to 7 (mean 5.06 ± 1.18). Affected brain regions, with some patients having overlapping lesions, included the frontal lobe (*N* = 5), temporal lobe (*N* = 3), parietal lobe (*N* = 3), and subcortical structures such as the basal ganglia (*N* = 3), thalamus (*N* = 2), cerebellum (*N* = 2), and brainstem (*N* = 1).

Figure 1a presents a schematic diagram of the eye-tracking experiment using a portable eye-tracking device. Figure 1b illustrates the process of freely observing familiar dramatic character faces. The red line segment overlaid on the person’s face simulates the scanpath during observation. The eye movement scanpaths of both a control subject and a stroke subject are shown across 10 trials in two different television series (see in Figure 1c).

**Figure 3:**
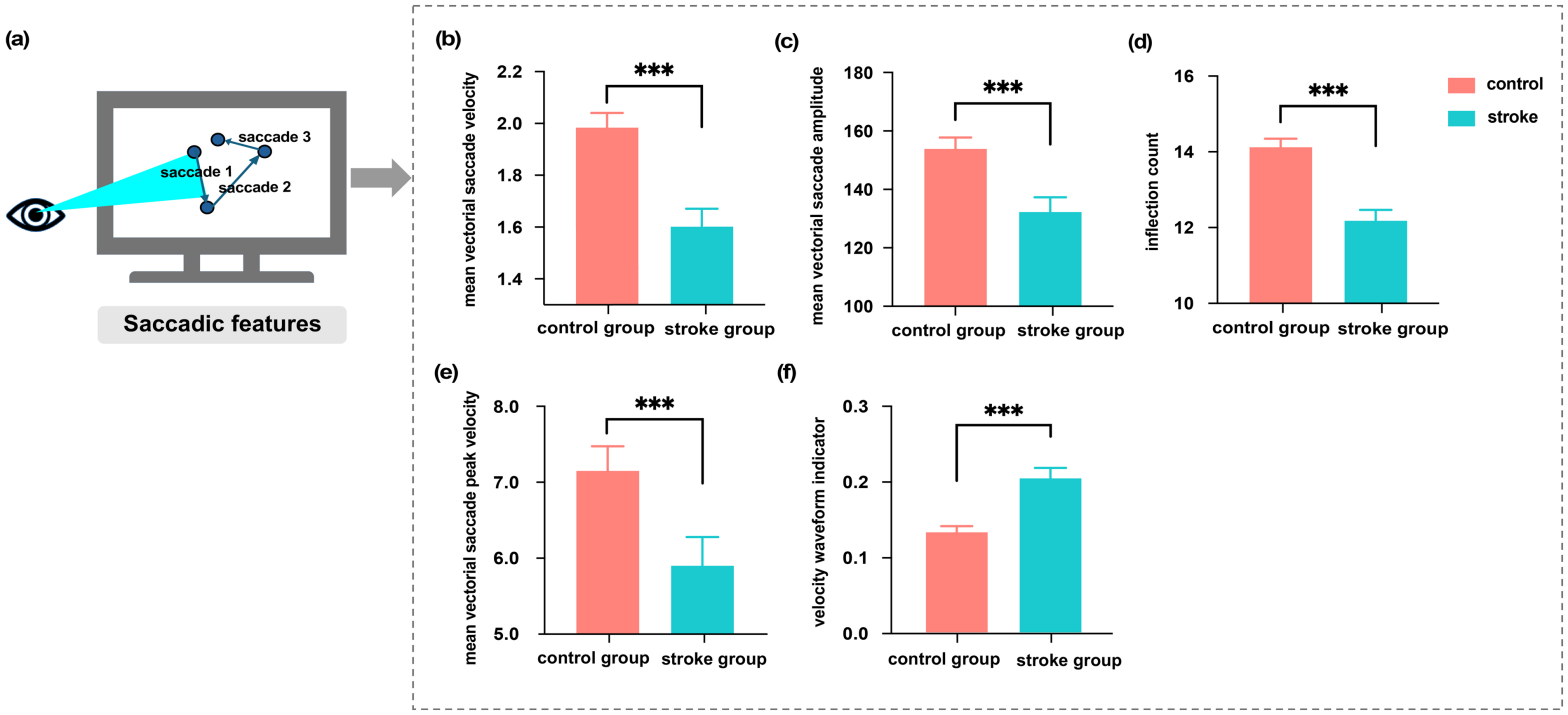
**(a)** Schematic illustration of the saccade process, with cyan shaded areas indicating the eye’s shift in attention to the target via a saccade. **(b–f)** Significant differences in saccadic features between healthy controls and stroke patients are shown as Mean ± SEM, with statistical significance indicated as *** (*p* < 0.001).

To elucidate group differences in eye movement features, we compared saccade, fixation, and scanpath features between stroke patients and healthy controls. Figure 3a illustrates the eye movement saccades, with the blue arrows on the screen indicating the process of saccades, which rapidly shift the focus of vision from one location to another. We first assessed the significant differences in saccadic features between the stroke and healthy groups (Figures 3b–f). We found that stroke patients exhibited a significant decrease in mean saccadic amplitude compared to healthy controls (132.46 ± 4.84 vs. 154.07 ± 3.69; z = −3.84, *p* < 0.001, *r* = 0.62, 95% CI [0.34, 0.79]), as well as slower mean saccade velocity (1.60 ± 0.07 vs. 1.99 ± 0.06; *z* = −5.28, *p* < 0.001, *r* = 0.85, 95% CI [0.58, 0.97]). Similarly, the inflection count was also lower in the stroke group (12.19 ± 0.27 vs. 14.13 ± 0.22; *z* = −5.55, *p* < 0.001, *r* = 0.89, 95% CI [0.63, 1.00]). Interestingly, mean vectorial saccade peak velocity was slower in patients (7.16 ± 0.32 vs. controls: 5.91 ± 0.37; *z* = −3.79, *p* < 0.001, *r* = 0.61, 95% CI [0.33, 0.78]); however, the velocity waveform indicator was significantly higher in stroke patients (0.22 ± 0.01 vs. controls: 0.14 ± 0.01; *z* = −3.56, *p* < 0.001, *r* = 0.57, 95% CI [0.29, 0.75]).

**Figure 4:**
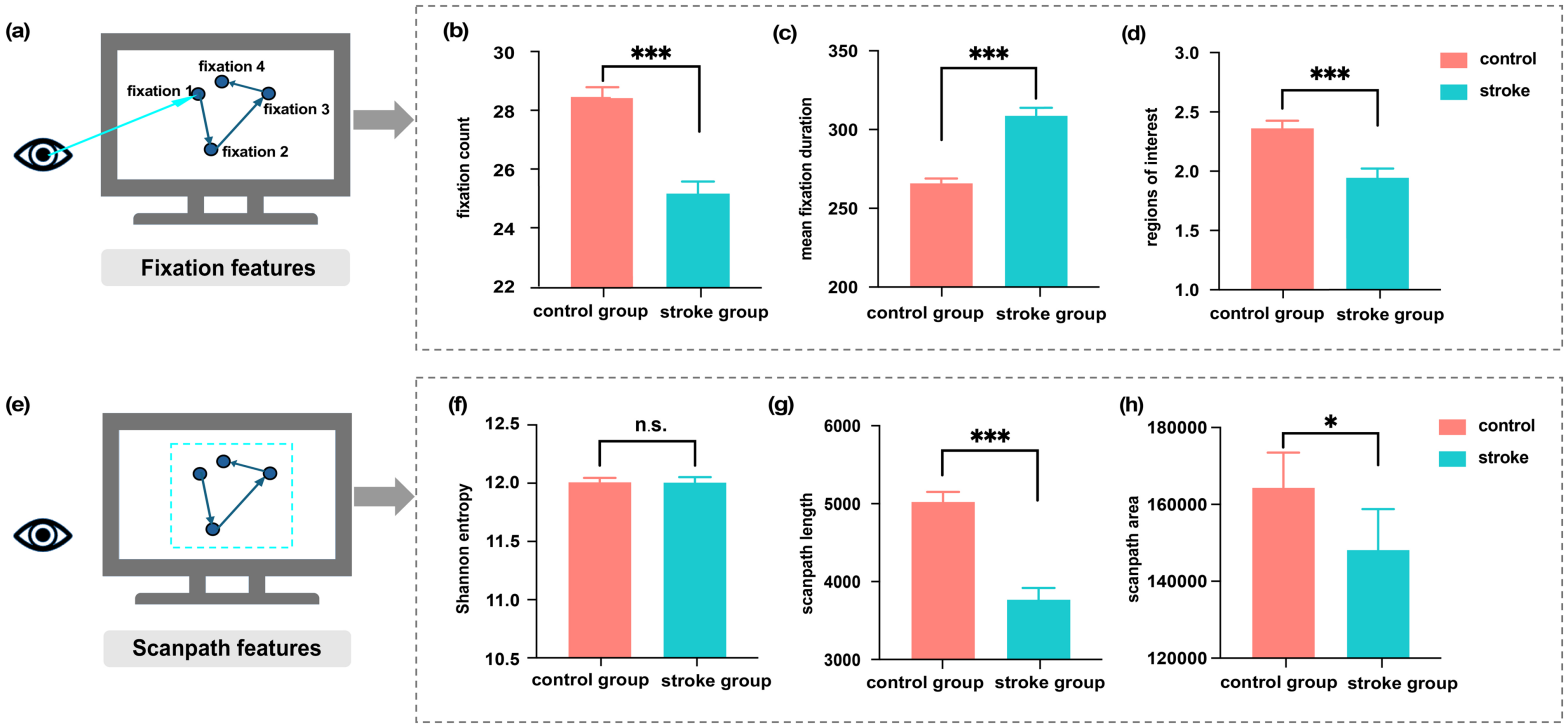
**(a)** Schematic illustration of the fixation process. **(b–d)** Significant differences in fixation features between healthy controls and stroke patients. **(e)** Schematic illustration of the overall scanpath. **(f–h)** Significant differences in scanpath features between healthy controls and stroke patients. All differences are shown as Mean ± SEM, with statistical significance indicated as n.s. (*p* > 0.05), * (*p* < 0.05), and *** (*p* < 0.001).

To gain further insight into eye movement dysfunctions in stroke patients, we compared the significant differences in fixation and scanpath features between the stroke and healthy groups. Figure 4a presents a schematic illustration of the fixation behavior, with cyan arrows indicating the eye’s targeting of important information (forming fixations) for detailed observation. Fixation features showed marked alterations in stroke group, as illustrated in Figures 4b–d. Specifically, mean fixation duration was prolonged in stroke patients (309.07 ± 4.77 *vs.* 266.16 ± 2.72; *z* = −7.96, *p* < 0.00, *r* > 0.99, 95% CI [0.99, 1.00]), who also displayed fewer fixation counts (25.24 ± 0.39 *vs.* 28.45 ± 0.32; *z* = −6.95, *p* < 0.001, *r* > 0.99, 95% CI [0.99, 1.00]) and explored fewer regions of interest (1.95 ± 0.07 *vs.* 2.37 ± 0.06; *z* = −4.54, *p* < 0.001, *r* = 0.73, 95% CI [0.45, 0.89]).

Figure 4e illustrates the overall scanpath, depicting the sequence of fixations and saccades that occur during the visual exploration of the stimulus. We assessed the significant differences in scanpath features between two groups (see in Figures 4f–h). Stroke patients performed shorter scanpath length (3774.34 ± 143.44 vs. 5029.59 ± 122.45; *z* = −6.68, *p* < 0.001, *r* > 0.99, 95% CI [0.99, 1.00]) and smaller scanpath areas (148220.66 ± 10552.67 vs. 164419.24 ± 9060.23; *z* = −2.00, *p* < 0.05, *r* = 0.32, 95% CI [0.04, 0.55]). Additionally, Shannon entropy did not differ significantly between groups (*t* (388) = 0.07, *p* = 0.94, Cohen’s d ≈ 0.007, 95% CI [−0.204, 0.218]).

### Group differences of intra-individual stability and inter-individual consistency

**Figure 5:**
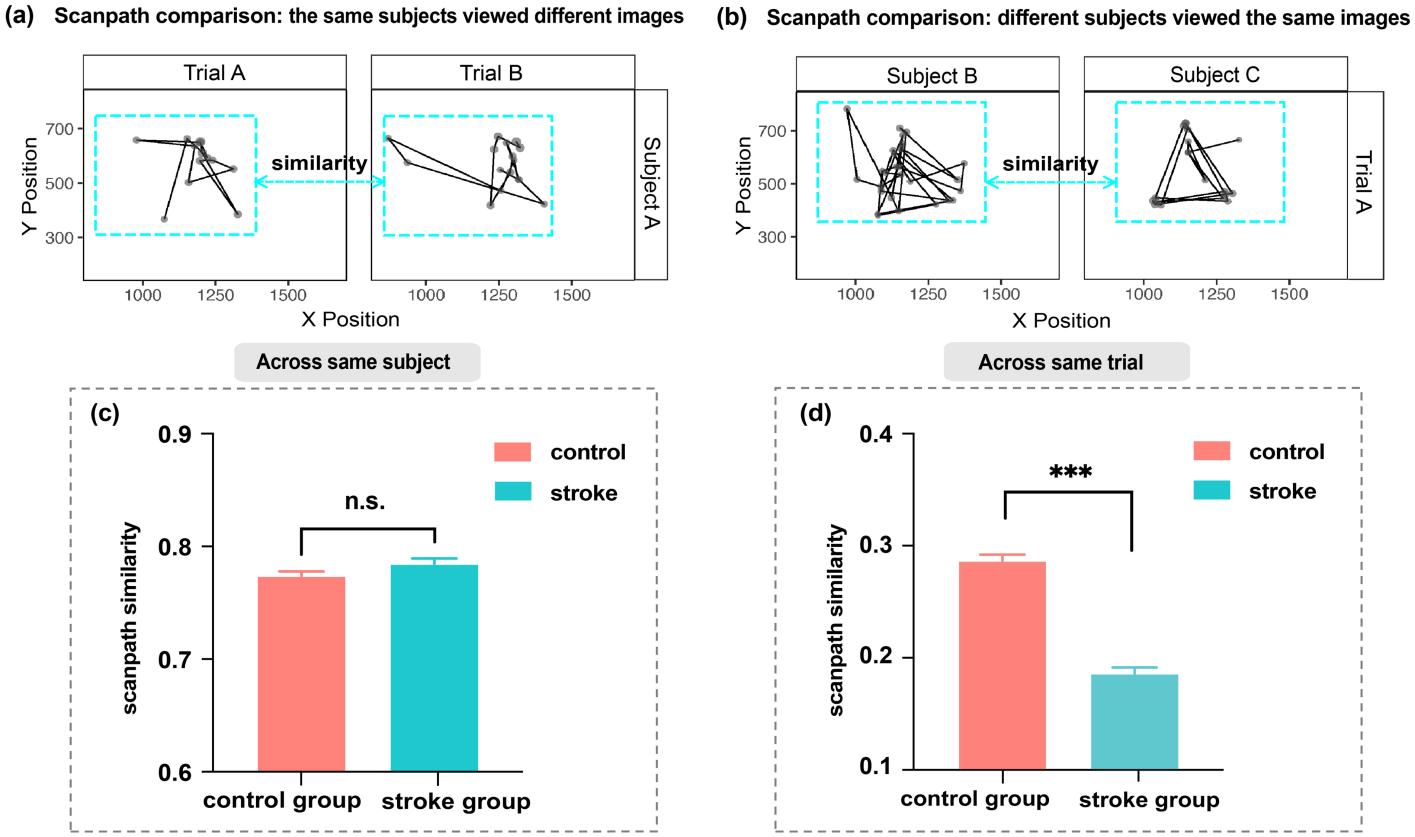
**(a)** The comparision of scanpaths of a subject on different trials. **(b)** The comparision of scanpaths of two subjects from the same group on the same trial. Black lines = scanpaths; dark grey dots = fixations (larger dots = longer duration). Scanpath similarity is calculated based on the comparison of two complete scanpaths (as illustrated in the cyan dashed box). **(c)** Scanpath similarity between stroke patients and healthy controls (approach from **a**). **(d)** Scanpath similarity between stroke patients and healthy controls (approach from **b**). All differences are shown as Mean ± SEM, with statistical significance indicated as n.s. (*p* > 0.05) and *** (*p* < 0.001).

Next, to further investigate the overall regularity of eye movement scanpaths, we conducted scanpath similarity analysis and compared differences between the stroke and control groups. This method is widely adopted in eye-tracking research to quantify visual exploration patterns, particularly in cognitive science (Mézière et al., 2024). We analyzed scanpath similarity through two directions: intra-individual stability (Figure 5a) and inter-individual consistency (Figure 5b). Figures 5c–d present two distinct measures of scanpath organization and the corresponding comparison results: (i) intra-individual stability, where both healthy controls (0.77) and stroke patients (0.78) showed comparable stability of individual scanpath patterns across varying stimuli(*z* = −1.88, *p* = 0.06); and (ii) inter-individual consistency, which revealed significantly lower scanpath similarity among stroke patients (0.19) compared to healthy controls (0.29; *z* = −14.64, *p* < 0.001), indicating reduced group-level pattern alignment when viewing identical images.

### Data reliability results

The AOI analysis confirmed that participants’ fixations were consistently concentrated on the core facial regions. The average proportion of fixation points (Figure 2b) falling inside the AOI was 88.50%, and the average proportion of total fixation time inside the AOI was 91.10% (Figure 2c). Specifically, 75% of trials had a fixation point proportion exceeding 85, and 95% of trials had a fixation time proportion above 85%. These consistently high values indicate that even without an explicit task, participants’ fixations were strongly concentrated on the central face areas. This pattern aligns with previous findings that viewers spontaneously direct their gaze to the eyes, nose, and mouth during free viewing of faces (Yarbus, 1967; Fletcher-Watson et al., 2008), supporting the validity of using a free viewing paradigm to study face processing behavior.

Moreover, the internal consistency analysis further confirmed the high reliability of oculomotor metrics across different trial subsets (see Supplementary Table 1 for full details). When using only the first 3 trials, ICCs ranged from 0.66 to 0.89, suggesting moderate to good agreement for key metrics even with a limited number of trials. Extending the analysis to the first 5 trials improved ICC values to between 0.81 and 0.96, indicating high reliability. With the first 7 trials, ICCs reached 0.95–0.97, demonstrating excellent consistency. These findings indicate that the oculomotor data robustly capture individual eye movement characteristics, even when derived from a reduced set of trials.

### Machine learning performance in stroke recognition via eye movement features

**Figure 6:**
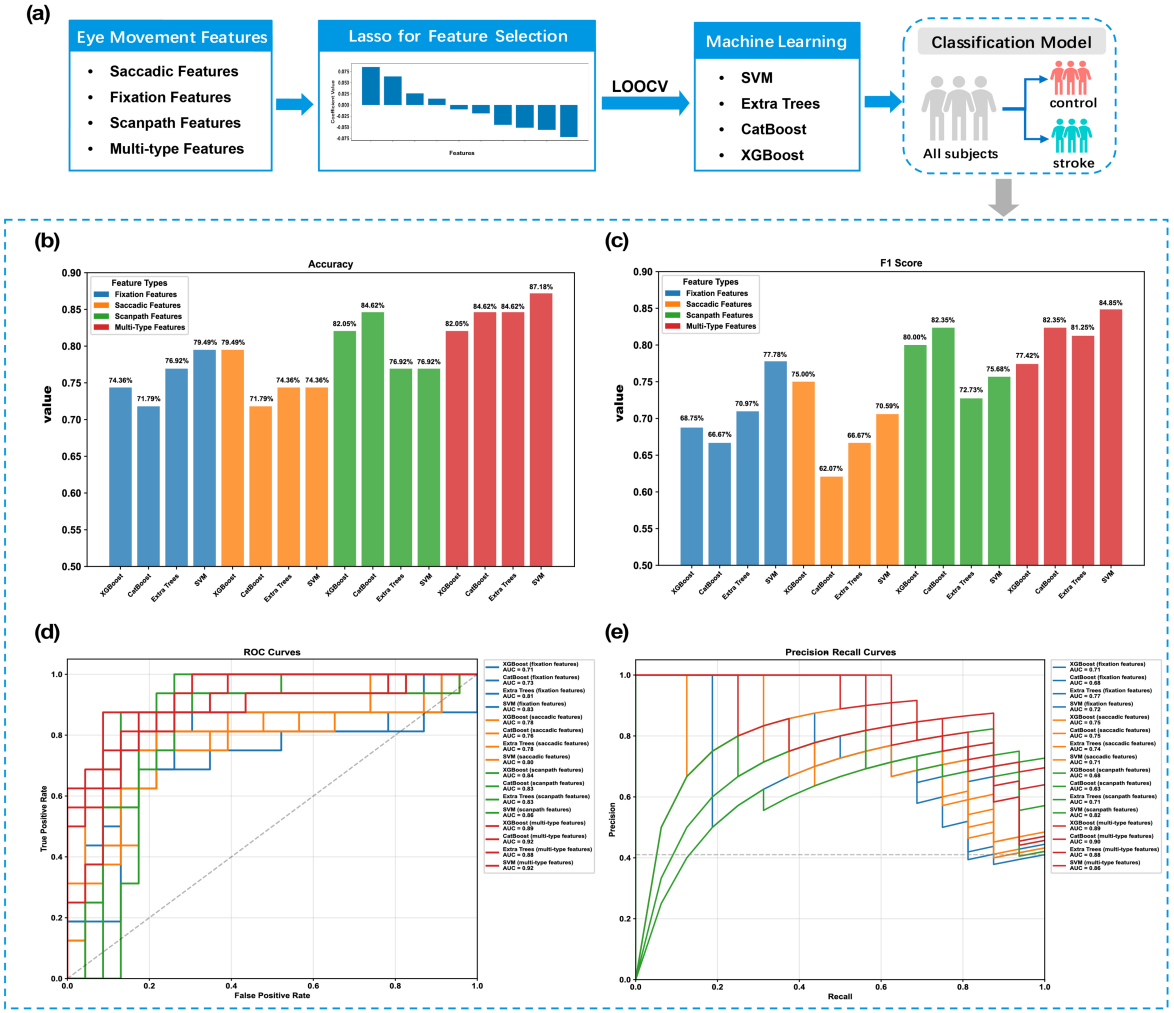
Machine learning workflow and classification performance for stroke identification. **(a)** The schematic representation of the analytical workflow, encompassing eye movement feature extraction, feature selection by Lasso, and machine learning for stroke recognition. **(b–e)** Comparative classification performance of the four employed machine learning algorithms.

To investigate whether eye movement features from stroke patient could precisely identify stroke, we generated machine learning models using single-type features, including saccade, fixation, and scanpath, as well as their combination. Figure 6a illustrates the analytical workflow, including feature extraction, feature selection by LASSO, and machine learning for stroke recognition. To ensure comparable feature representation across categories, we applied standardized LASSO-regularized feature selection, determining the optimal number of features as 10 across all categories.

Figures 6b–e present the mean values of accuracy, F1-score, AUROC, and AUCPR obtained through LOOCV. Among the feature sets, Saccadic features showed the lowest performance. XGBoost achieved the best performance on saccadic features, with an accuracy of 79.49% and an F1-score of 75.00%. Both the SVM and Extra Trees models exhibited an accuracy of 74.36%, while the CatBoost model performed slightly less effectively with these features. In contrast, models trained on fixation features showed that the SVM model demonstrated superior classification capability, achieving the highest accuracy of 79.49% and the highest F1-score of 77.78%. The Extra Trees model also displayed a competitively high accuracy of 76.92%; however, its lower F1-score of 70.97% indicated potential limitations in classification balance when relying solely on fixation information.

Scanpath features exhibited significantly greater discriminative power compared to localized features such as saccades and fixations. The performance results revealed that the CatBoost model achieved the highest accuracy of 84.62% and F1-score of 82.35% within this feature set. XGBoost also demonstrated robust performance, with an accuracy of 82.05% and an F1-score of 80%. Furthermore, the SVM model attained the highest AUROC score of 0.86 for these features, underscoring the strong discriminatory capability of combining scanpath features with SVM.

The multi-type features, which include saccade, fixation, and scanpath characteristics, demonstrated enhancements across all classifiers, resulting in significant improvements compared to models that solely employ scanpath features. The SVM model exhibited the best overall performance, attaining peak results across all evaluated metrics. By incorporating these multi-dimensional oculomotor features, our machine learning model achieved a high accuracy of 87.18% and an AUROC of 0.92 in differentiating stroke patients. Both the CatBoost and Extra Trees models also demonstrated strong accuracy at 84.62%, while optimizing AUROC and AUCPR values through feature fusion. Notably, the CatBoost model achieved the highest AUCPR score of 0.90 among all model-feature combinations. The consistently high AUCPR scores, ranging from 0.86 to 0.90, robustly validate that the multi-type feature strategy, which integrates saccadic, fixation, and scanpath information, provides a more comprehensive and stable representation of cognitive states than any single feature type, ensuring strong class separation capability across varying decision thresholds.

## Discussion

Stroke is a significant chronic non-communicable disease that poses a serious threat to human health, being the second leading cause of death and the primary cause of disability worldwide (Yanez et al., 2020). Neuroimaging techniques such as CT and MRI, while remaining the diagnostic gold standard of stroke, face three major limitations: limited accessibility outside tertiary care centers, inability for continuous monitoring, and poor scalability for mass screening (Sahiner et al., 2017). Given the vast number of stroke patients and the potential at-risk population, there is an urgent need for portable, cost-effective screening tools capable of providing preliminary neurological evaluations. Here, we present a method leveraging portable eye-tracking technology to identify stroke-induced oculomotor abnormalities. This approach enables rapid evaluation without the need for complex infrastructure or highly trained personnel. Importantly, it holds significant potential for early screening in resource-limited settings, which could facilitate prompt intervention that may reduce irreversible neurological damage, decrease long-term disability, and lower mortality rates (de la Ossa et al., 2008).

In this study, we used eye-tracking to analyze eye movement features during free viewing of famous faces. We found distinct differences in oculomotor behavior between stroke patients and healthy controls. Stroke patients had reduced saccade amplitude and velocity, aligning with prior research on impaired saccadic control post-stroke (Kremmyda et al., 2011). Chan et al. also showed that cognitively impaired stroke patients had slower saccadic velocity than those without cognitive deficits (Chan et al., 2023). This suggests that reduced saccade velocity in stroke patients may result from disruptions in both saccadic control and cognitive processing. Interestingly, while stroke patients had slower mean and peak saccade velocities compared to controls, they showed higher velocity waveform indicators. This paradox may indicate compensatory neural adaptations or impaired saccadic control leading to abrupt eye movements (Catz et al., 1997).

Additionally, Teslenko et al. (2018)’s study demonstrated that brain damage affects fixation stability and impairs the shift of fixation. Consistent with this finding, our results revealed altered fixation patterns in stroke patients, including prolonged fixation duration and decreased fixation frequency, consistent with previous studies. These changes may indicate either impaired attentional disengagement or an adaptive strategy to cope with complex visual stimuli. Oculomotor changes observed in stroke patients are associated with restricted visual exploration, characterized by fewer regions of interest and a shorter scanpath length. The decrease in the regions of interest indicates a potential narrowing of the attentional spotlight or a delay in disengaging from salient features (Corbetta and Shulman, 2002). The shortened scanpath length in stroke patients primarily reflects impaired active exploration capabilities and simplified search strategies, which may arise from either structural damage to neural networks or compensatory mechanisms arising from insufficient cognitive resources (Rowe et al., 2019). These findings are in line with previous studies on visuospatial deficits following stroke (Elfeky et al., 2021). Overall, our findings reveal how oculomotor features associated with stroke present across different features, with a convergence of characteristics like reduced saccade velocity, prolonged fixation duration, and restricted visual exploration, all reflecting underlying neural deficits. These insights offer a basis for constructing eye-tracking biomarkers for stroke diagnosis.

Our findings also shed light on the characteristics of intra-individual stability and inter-individual consistency in the stroke group compared to healthy controls. Intra-individual stability was similar between groups, indicating that stroke does not disrupt individual exploration patterns, aligning with Scanpath Theory’s preserved idiosyncratic patterns when viewing familiar stimuli (Noton and Stark, 1971). In contrast, stroke patients exhibited significantly lower inter-individual scanpath similarity, which may indicate disrupted shared attentional patterns due to lesion-induced dysfunction (Sperber et al., 2024). Additionally, lesion heterogeneity across individuals could also play a role, as variations in lesion location may differentially affect neural circuits involved in attention and eye movement control, such as frontoparietal and subcortical networks (Sperber et al., 2024; Scolari et al., 2015; Mustin et al., 2024). However, healthy controls demonstrated higher inter-individual scanpath similarity, reflecting conserved attentional biases toward salient features, such as the eyes and nose (Tomasello et al., 2007). Thus, while stroke appears to preserve intra-individual stability in eye movements, it impairs inter-individual consistency.

Another essential finding in the current study is that eye-tracking during free visual exploration of familiar faces yields highly discriminative features for stroke recognition. The integrated feature set outperformed single-category features, with Extra Trees showing notable gains in accuracy (from 74.36 to 84.62%) and F1-score (from 72.73 to 81.25%). SVM achieved a high accuracy (87.18%) and an AUROC value (0.92), significantly improving on existing methods. These results highlight the benefits of combining fixation, saccadic, and scanpath features, supporting that multi-type eye movement features enhance diagnostic model performance. Unlike prior VR-based studies with features of fixation frequency and duration that showed modest performance (AUC = 0.64) (Brouwer et al., 2022), our approach likely benefits from multi-type features to better capture stroke-related eye movement dysfunction. The high AUCPR scores (0.86–0.90) across models confirm robust class separability, validating the effectiveness of integrating a diverse range of eye movement features. Our findings align with previous studies (Wu et al., 2024) that have highlighted the limitations of relying solely on single-type features for balanced classification tasks, particularly in clinical scenarios requiring optimized sensitivity and specificity. While our machine learning model demonstrates promising results that warrant further investigation, its exploratory nature and the limitations of our sample size prevent it from informing clinical practice at this stage. It is essential to validate the model’s performance in a larger, independent population to ensure its applicability in clinical settings.

Our findings have limited generalizability owing to two key limitations. First, the stimuli were derived from a single cultural context. Differences in cultural familiarity with the stimuli may modulate these attention allocation patterns in cross-cultural studies (Goh et al., 2009; Ueda and Komiya, 2012; Blais et al., 2008). Therefore, our findings must be interpreted within this specific cultural framework. Future studies should incorporate cross-culturally designed stimulus sets and recruit participants from diverse cultural backgrounds to validate the generalizability of the current findings and extend their applicability. Second, the limited sample size in our study constrained the depth of the group-level comparison between stroke patients and healthy controls, preventing a comprehensive capture of the variability arising from different stroke types and lesion locations. Future studies should prioritize larger, multi-center cohorts to validate these preliminary findings and dissect the contributions of lesion topology and severity to eye movement variability. Despite these limitations, our findings provide preliminary evidence supporting the significant potential of eye-tracking during free visual exploration of familiar dramatic character faces as a scalable and cost-effective approach for rapid stroke recognition, which could ultimately contribute to improved patient outcomes and reduced healthcare burdens.

## Conclusion

In summary, our study shows that eye-tracking during the FVE task with familiar dramatic character faces is effective for rapid and accurate stroke recognition. This approach is non-invasive, requires only a brief testing period, and yields diagnostically informative data. The integration of fixation, saccadic, and scanpath features enhanced model performance, stability, and interpretability. We emphasize that our approach is to serve as a complementary screening tool in community or prehospital settings with limited resources. Future work should replicate these findings in larger, diverse cohorts and explore integrating other diagnostic methods to improve the accuracy and reliability of stroke recognition, facilitating timely rehabilitative interventions following a stroke.

## Data Availability

The original contributions presented in the study are included in the article/Supplementary material, further inquiries can be directed to the corresponding author.
