# Supplementary material for: Eye-tracking during free visual exploration of familiar dramatic character faces facilitates rapid and accurate stroke recognition
Source: Front Neurosci. 2026 Jan 6;19:1692719. doi: 10.3389/fnins.2025.1692719 (PMC12816275; doi:10.3389/fnins.2025.1692719)
Supplement: Supplementary file 1 [file Table_1.DOCX]

**Supplement Materials**

**Supplementary Tables**

The analysis revealed that with only the first 3 trials, the intraclass correlation coefficients (ICCs) ranged from 0.66 to 0.89, indicating good agreement for key metrics even with fewer trials (**Supplementary Table 1**). With the first 5 trials, ICC values improved to between 0.81 and 0.96. With the first 7 trials, ICCs reached 0.95–0.97, demonstrating high consistency. These results confirm that although the number of trials in this study was limited to 10, the data obtained reliably capture participants’ eye‑movement characteristics.

**Supplementary Table 1. Intraclass Correlation Coefficient of Oculomotor Parameters at Different Numbers of Trials.**

| **Feature** | **fixation count** | **mean fixation duration** | **regions of interest** | **mean vectorial saccade amplitude** | **mean vectorial saccade velocity** | **inflection count** | **mean vectorial saccade peak velocity** | **velocity waveform indicator** | **Shannon entropy** | **scanpath length** | **scanpath area** |
| --- | --- | --- | --- | --- | --- | --- | --- | --- | --- | --- | --- |
| **ICC_3_trials** | 0.89 | 0.84 | 0.85 | 0.77 | 0.79 | 0.86 | 0.80 | 0.81 | 0.66 | 0.77 | 0.71 |
| **ICC_5_trials** | 0.96 | 0.94 | 0.94 | 0.93 | 0.92 | 0.90 | 0.91 | 0.90 | 0.90 | 0.86 | 0.81 |
| **ICC_7_trials** | 0.97 | 0.97 | 0.96 | 0.96 | 0.95 | 0.96 | 0.95 | 0.96 | 0.94 | 0.94 | 0.95 |

**Supplementary Table 2** presents the mean values of accuracy, F1-score, AUCROC, and AUCPR derived from repeated stratified 5-fold cross validation, respectively. Saccadic features showed the lowest and least stable discriminative performance. SVM achieved the highest accuracy (77.93%), closely followed by Extra Trees (77.29%), with F1-scores ranging from 62.81% to 66.72%, indicating challenges in maintaining precision-recall balance. Among the evaluated models by fixation features, SVM exhibited optimal performance (accuracy: 77.86%; AUCROC: 0.87), while Extra Trees achieved comparable accuracy (76.57%). The limited range of F1-scores (64.70%-70.67%) and the moderate performance ceiling (peak accuracy < 78%) highlight the limitations of relying solely on fixation features for balanced classification tasks. Furthermore, scanpath features consistently outperformed both fixation and saccadic features across all evaluation metrics. XGBoost achieved the highest accuracy (82.21%) and F1-score (77.81%), while SVM exhibited the most balanced performance profile (accuracy: 79.93%; AUCPR: 0.88). Notably, Extra Trees and SVM showed strong discriminative capability, achieving AUCROC values above 0.90, with Extra Trees attaining a high AUCPR (0.8498).

The LASSO-selected multi-type features, encompassing saccade, fixation, and scanpath features, achieved improvements across all classifiers. SVM demonstrated optimal performance (accuracy: 87.36%; AUCROC: 0.94). Extra Trees exhibited robust performance as the secondary choice (accuracy: 88.29%; AUCROC: 0.9174). CatBoost displayed moderate accuracy (79.07%) with consistent F1-score (72.68%) and AUCPR (0.88). The consistently high AUCPR scores across models (0.88-0.90) confirm that the selected features provide robust class separability across different decision thresholds.

**Supplementary Table 2. Model performance evaluated by 5-fold CV.**

| Feature Set | Algorithm | AUROC (Mean) | AUCPR (Mean) | Accuracy (Mean) | F1 Score (Mean) |
| --- | --- | --- | --- | --- | --- |
| fixation features | XGBoost | 0.74 | 0.76 | 71.86% | 66.19% |
| fixation features | CatBoost | 0.79 | 0.82 | 70.93% | 64.70% |
| fixation features | Extra Trees | 0.85 | 0.84 | 76.57% | 69.95% |
| fixation features | SVM | 0.91 | 0.91 | 77.86% | 70.67% |
| saccadic features | XGBoost | 0.79 | 0.82 | 73.21% | 65.17% |
| saccadic features | CatBoost | 0.75 | 0.76 | 73.71% | 66.72% |
| saccadic features | Extra Trees | 0.85 | 0.85 | 77.29% | 63.62% |
| saccadic features | SVM | 0.85 | 0.86 | 77.93% | 63.43% |
| scanpath features | XGBoost | 0.89 | 0.83 | 82.21% | 77.81% |
| scanpath features | CatBoost | 0.87 | 0.86 | 78.57% | 72.90% |
| scanpath features | Extra Trees | 0.93 | 0.92 | 79.14% | 71.47% |
| scanpath features | SVM | 0.91 | 0.92 | 79.93% | 75.10% |
| multi-type features | XGBoost | 0.91 | 0.91 | 82.00% | 76.30% |
| multi-type features | CatBoost | 0.92 | 0.92 | 79.07% | 72.68% |
| multi-type features | Extra Trees | 0.94 | 0.94 | 88.29% | 85.51% |
| multi-type features | SVM | 0.95 | 0.95 | 87.36% | 83.92% |
